# Gut microbiota-generated short-chain fatty acids are involved in para-chlorophenylalanine-induced cognitive disorders

**DOI:** 10.3389/fmicb.2022.1028913

**Published:** 2022-11-07

**Authors:** Yanbo Liu, Zhen Li, Tianning Sun, Zhigang He, Hongbing Xiang, Jun Xiong

**Affiliations:** ^1^Department of Anesthesiology and Pain Medicine, Tongji Hospital, Tongji Medical College, Huazhong University of Science and Technology, Wuhan, China; ^2^Hepatobiliary Surgery Center, Union Hospital, Tongji Medical College, Huazhong University of Science and Technology, Wuhan, China

**Keywords:** gut microbiota, short-chain fatty acids (SCFAs), para-chlorophenylalanine (PCPA), cognitive disorders, dysbiosis

## Abstract

Neurocognitive disorders (NCDs) include complex and multifactorial diseases that affect many patients. The 5-hydroxytryptamine (5-HT) neuron system plays an important role in NCDs. Existing studies have reported that para-chlorophenylalanine (PCPA), a 5-HT scavenger, has a negative effect on cognitive function. However, we believe that PCPA may result in NCDs through other pathways. To explore this possibility, behavioral tests were performed to evaluate the cognitive function of PCPA-treated mice, suggesting the appearance of cognitive dysfunction and depression-like behavior. Furthermore, 16S rRNA and metabolomic analyses revealed that dysbiosis and acetate alternation could be related to PCPA-induced NCDs. Our results suggest that not only 5-HT depletion but also dysbiosis and acetate alternation contributed to PCPA-related NCDs. Specifically, the latter promotes NCDs by reducing short-chain fatty acid levels. Together, these findings provide an alternative perspective on PCPA-induced NCDs.

## Introduction

Neurocognitive disorders are a group of the most common mental disorders associated with an increased incidence of conditions such as Alzheimer’s disease (Liu et al., 2022a) and Parkinson’s disease (Baba et al., 2022). According to the Diagnostic and Statistical Manual of Mental Disorders, Fifth Edition, NCDs often begin with delirium, followed by major syndromes and mild NCDs as well as their etiological subtypes, in which the underlying pathology is potentially determined (American Psychiatric Publication, 2013).

Several studies on NCDs have attempted to outline its potential mechanisms, and theories about the relationship between 5-HT neurons and cognition have been the core of some of the most active controversies in neuroscience research (Meneses, 1999; Bacqué-Cazenave et al., 2020; Brownlow et al., 2020; Ni et al., 2021). Given the negative effects of NCDs on individuals and their correlation with other outcomes related to the social burden, this emphasis is understandable. 5-HT neurons play a key role in the ascending reticular activating system; therefore, they are related to the regulation of sleep–wake behavior (Mccormick and Bal, 1997; Monti, 2011; Ito et al., 2013). Para-chlorophenylalanine (PCPA), an irreversible inhibitor of tryptophan hydroxylase, can pharmacologically deplete 5-HT and result in insomnia in animal models (Mouret et al., 1968; Jouvet, 1969; Murray et al., 2015). Previous studies have reported that PCPA can cause insomnia (Mouret et al., 1968) and anxiety (Farabollini et al., 1988) in animals. Most present studies have focused on the effects of 5-HT depletion caused by PCPA and subsequent NCDs (Pham et al., 2017; Riga et al., 2020; Ghaheri et al., 2022). However, it is unknown whether PCPA exerts its effect through other pathways.

The gut microbiota plays a crucial role in numerous host physiological processes and the development of various diseases (Li et al., 2020; Si et al., 2022b; Zhou et al., 2022a). Many studies have identified the relationship between gut microbiota and NCDs (Arnoriaga-Rodríguez and Fernández-Real, 2019; Liu et al., 2020; Wang et al., 2021). For example, a study revealed that changes in the intestinal microbiota affect the blood–brain barrier through gut-derived neurotransmitters and microbial metabolites, which can be associated with various cognitive disorders (Arnoriaga-Rodríguez and Fernández-Real, 2019). Short-chain fatty acids (SCFAs), such as acetate, propionate, and butyrate, are the primary producers of dietary fiber, which is produced through saccharolytic fermentation in the gastrointestinal tract. SCFAs affect the cognitive function of the host through local effects, immune system, and gut-brain signaling (Liu et al., 2020). Furthermore, SCFAs can act by regulating neurotransmitters. In previous studies, propionate induced the gene transcription of tyrosine hydroxylase, an enzyme involved in catecholamine biosynthesis (Nankova et al., 2014; Dalile et al., 2019), which acts as a neurotransmitter in the center neuron system. Yao et al. (2022) and Si et al. (2022a) demonstrated a relationship between PCPA administration and dysbiosis in animals. Especially, the latter study showed that PCPA-induced 5-HT depletion affects gut microbiota in rodents. These findings suggest that the gut microbiota and its main producers act as important factors involved in the progression of PCPA-induced NCDs.

Based on the background mentioned above, we performed extensive research on cognition dysfunction caused by PCPA. After treatment with PCPA, behavioral tests, 16S ribosomal RNA (16S rRNA) analysis, and serum 5-HT and SCFA level assessment were conducted. Our results indicate that PCPA-induced NCDs are associated with dysbiosis through SCFAs.

## Materials and methods

### Animals

The assays were accomplished with Male C57BL/6J mice, 6–8 weeks of age, procured from the Beijing Vital River Laboratory Animal Technology Co., Ltd., (Beijing, China). They were kept in standard housing conditions (a regimen of 23 ± 1°C room temperature with a relative humidity of 55 ± 5% and a 12/12 h light/dark cycle). The Animal Care and Use Committee of Tongji Hospital, Tongji Medical College approved the current study (no. TJ0803).

### Para-chlorophenylalanine treatment

Para-chlorophenylalanine (C6506, Sigma Co., Ltd.) was insoluble in PBS, saline, or other delivery vehicles (Murray et al., 2015). Thus, PCPA mice were administrated using PCPA methyl ester (C3635, Sigma Co., Ltd.) intraperitoneal injection. The impact of PCPA methyl ester on the 5-HT level had been proved by previous studies (Du Jardin et al., 2017; Narasingam et al., 2017). The dose administered was 300 mg/kg daily to the PCPA group for five consecutive days (Wang et al., 2020). PCPA was dissolved in PBS and the vehicle group received volume-matched PBS injections. After treatment, behavioral tests were performed to determine the cognitive function of the treated individuals.

### Behavior test

Open field test (OFT), novel object recognition test (NORT), and Y-maze test (Y-Maze) were undergone to assess cognitive impairment. Behavioral data were automatically analyzed through an intelligent video tracking system.

#### Open field test

As previously described (Li et al., 2022b; Yang et al., 2022), the mice were kept in the center of a gray polyethylene box with an open field chamber (L × W × H: 40 cm × 40 cm × 40 cm). They moved freely under dim light (300 lux) for 5 min, and the total distance traveled, motion time, and speed were analyzed.

#### Novel object recognition test

The NORT test was implemented as previously described (Antunes and Biala, 2012; Li et al., 2021). After the phase of environmental adaption, which allowed mice to explore the open field freely for 5 min, two identical objects were placed in an open field at two corners, 6 cm away from each border. In the first stage, the animal was allowed to conduct a free exploration for 5 min, and the total exploration time around each object was recorded. During the second stage, a similar experiment was performed, except that one of the two objects was replaced by a novel object with the same size but a different appearance. The exploration time was recorded around the novel (NT) and familiar objects (FT). Recognition Index $=\frac{\left(\text{NT}-\text{FT}\right)}{\left(\text{NT}+\text{FT}\right)}$. After each experiment, the apparatus was wiped with 75% ethanol to eliminate odor.

#### Y-maze

As previously described (Yang et al., 2022), the Y-maze device was made of gray polyethylene and had three arms, each at an angle of 120° (L × W × H: 30 cm × 8 cm × 15 cm). The three arms were randomly assigned: the initial arm for the initial animal to be placed and to begin exploring (always open), the new arm (blocked during the first trial but opened in the second one), and the other arm (always open). In the first stage, the mice were kept in the starting arm (the new arm was blocked), allowed to explore for 10 min, and ultimately returned to the cage. After 2 h, the second stage was performed in which the mice were kept in the same starting arm (with the new one open) and allowed to explore the maze for 5 min. We recorded the time spent within each arm, the number of mice entering the new arm, and the total number and the movement distance of the mice for analysis.

### Tissue sample collection

#### Fecal samples

Fecal samples were obtained using the method detailed previously (Zhang et al., 2022). The mice were kept in a clean cage using sterile paper at the bottom. Fecal samples were collected immediately after defecating inside a sterilized centrifuge tube. The filter paper was changed after each mouse. Then, fecal samples were stored inside a −80°C freezer. Then, 16S rRNA gene sequencing of fecal samples was performed at OEBiotech Co., Ltd., (Shanghai, China). DNA extraction was carried out using the DNA extraction kit (Tiangen Biotechnology Co., Ltd., Beijing, China). V3–V4 (or V4–V5) variable regions of 16S rRNA genes were amplified with universal primers 343 F and 798 R. Finally, the PCR products were purified for further sequencing. Clean reads were subjected to primer sequence removal and clustering to generate operational taxonomic units (OTUs) through the Vsearch software with a 97% similarity cutoff. All representative reads were annotated and blasted using the Silva database version 123 (or Greengens) through the RDP classifier (confidence threshold was 70%). All representative reads were annotated and blasted against the Unite database (ITSs rDNA).

#### Serum and hippocampus samples

The mice were anesthetized through intraperitoneal injection of 1% sodium pentobarbital (0.01 ml/g) and the eyeball was removed to obtain the blood samples. After centrifugation, the serum was stored in a −80°C freezer to perform biochemical analysis. The hippocampus was carefully dissected from the brain and kept at −80°C. The biochemical and histological analyses were done in the Wuhan Servicebio Technology Co., Ltd.

### Enzyme-linked immunosorbent assay

The levels of 5-hydroxytryptamine (5-HT) in the serum of mice were determined using the corresponding ELISA kit (Bio-Swamp Co., Ltd.). The whole process was carried out following the manufacturer’s protocol.

### Determination of shorty chain fatty acids

Briefly, 30 mg accurately weighed samples were treated using methanol and L-2-chlorophenylalanine after crushing and homogenization. The ACQUITY UPLC I-Class system (Waters Corporation, Milford, CT, USA) and VION IMS QTOF Mass spectrometer (Waters Corporation, Milford, CT, USA) were used to determine the metabolic profiling in both ESI positive and ESI negative ion modes. The original LC-MS data were processed using the software Progenesis QI V2.3 (Non-linear, Dynamics, Newcastle, UK) to filter baseline, identify peaks, integral, retention time correction, peak alignment, and normalization. Then, the principle component analysis and orthogonal partial least-squares-discriminant analysis were performed using R to analyze the correlation between the two groups.

### Pentobarbital-induced sleep test

According to previous studies (Wang et al., 2020; Kim et al., 2021), sodium pentobarbital i.p. (25 mg/kg) was administrated after 30 min of the last administration. The state of sleep obtained was defined as the loss of righting reflex for more than 1 min. The percentage of sleep onset was calculated as follows: Sleep onset (%) = (no. of falling asleep/total no.) × 100%

### Statistical analysis

All the quantification data were expressed as means ± SEM, with error bars representing SEM. Statistical analyses were undergone using the SPSS software version 25.0 (SPSS Inc., Armonk, New York, NY, USA) and the GraphPad Prism software (GraphPad Prism Software, Inc.). Behavioral tests were analyzed through one-way or two-way analysis of variance (ANOVA), followed by *post hoc* Bonferroni’s test. *P* < 0.05 was considered statistically significant.

## Results

### Para-chlorophenylalanine treatment significantly depleted serum 5-hydroxytryptamine in mice

**FIGURE 1:**
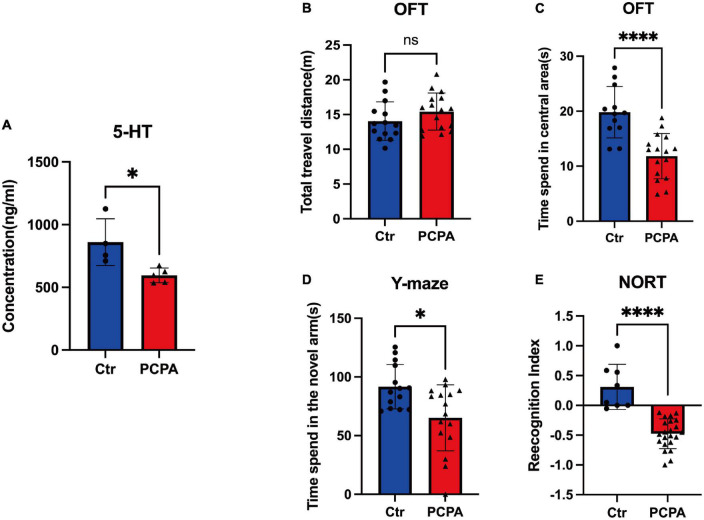
Para-chlorophenylalanine (PCPA) treatment induced cognitive dysfunction and depression-like behavior in mice. **(A)** Concentration. **(B)** Total travel distance of open field test (OFT). **(C)** Time spent in the central area of OFT. **(D)** Time spent in the novel arm of the Y-maze test. **(E)** Recognition index of novel object recognition test (NORT). Data are presented as the mean ± SEM. *P < 0.05; ****P < 0.0001; ns, not significant.

We first compared 5-HT levels between animals in the control group and the PCPA group to examine the depletion effect on blood 5-HT under PCPA [300 mg/kg, intraperitoneal injection (i.p.)]. The comparison depicted significant differences (Figure 1A), consistent with the previous studies (Farabollini et al., 1988; Zhou et al., 2022b).

### Para-chlorophenylalanine-treated mice displayed cognitive behavioral deficits

We performed behavioral tests between the two groups to better understand the cognitive change caused by PCPA. Compared with the control group, the time spent in the central area of the PCPA group reduced significantly (Figure 1B). However, there was no difference between the two groups (Figure 1C). Based on the Y-maze test, the PCPA group tended to spend less time in the novel arm than the control group (Figure 1D). Simultaneously, the recognition index (RI) of the PCPA group was significantly reduced (Figure 1E).

### Para-chlorophenylalanine treatment induced dysbiosis of gut microbiota in mice

**FIGURE 2:**
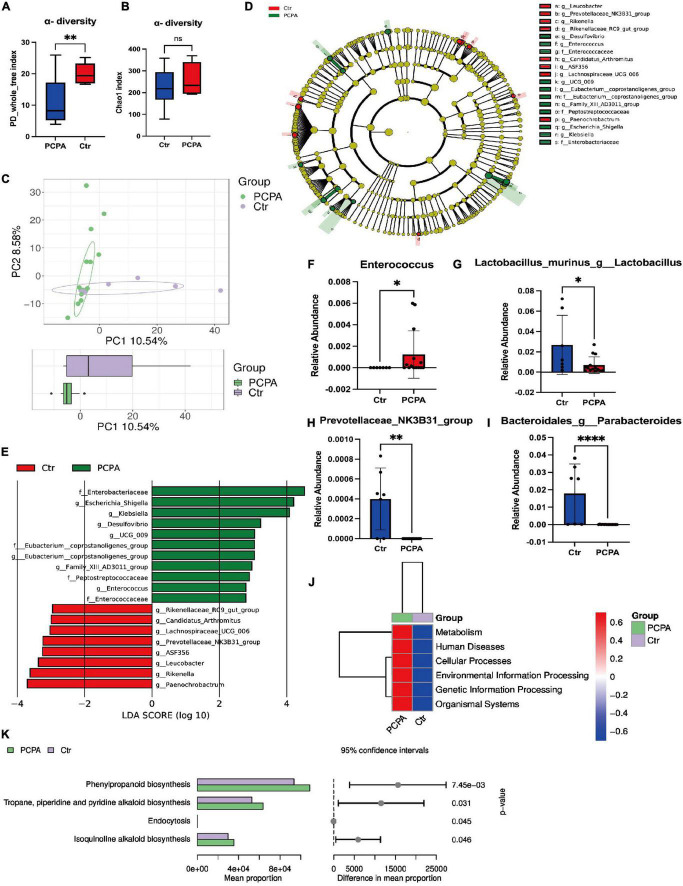
Para-chlorophenylalanine (PCPA) treatment induced gut microbiota alternation in mice. **(A)** α-diversity Chao1 index. **(B)** α-diversity PD Whole Tree Index. **(C)** Boxplot of principal component analysis (PCA). **(D,E)** Linear discriminant analysis Effect Size (LEfSe) analysis. **(F)** Relative abundance of the species g_Parabacteroides. **(G)** Relative abundance of the species g_Lactobacillus. **(H)** Relative abundance of the species g_Escherichia-Shigella. **(I)** Relative abundance of the family Enterobacteriaceae. **(J)** Relative abundance of the family Peptostreptococcaceae. **(K)** Relative abundance of the family Enterococcaceae. **(L–N)** KEGG analysis. Data are presented as the mean ± SEM. *P < 0.05; *P < 0.01; ^****^P < 0.0001; ns, not significant.

We pooled fresh feces from the two groups and performed 16S rRNA gene sequencing for fecal microbiota to figure out the community structure and diversity of the gut microbiota. As depicted in Figure 2, PD Whole Tree Index revealed that control mice had more gut microbial diversities than PCPA mice (Figure 2A). However, there was no difference in the Chao1 index between the two groups (Figure 2B). For Beta diversity, the principal component analysis (PCA) depicted the significant compositional discriminations inside the gut microbiota of the two groups (Figure 2C).

In addition, PCPA-associated bacterial taxa were evaluated by differential abundance analysis. Based on the linear discriminant analysis effect size (LEfSe) analysis, 19 discriminative features were identified (Figures 2D,E). Next, a statistical analysis of the alternations in relative abundances of fecal microbiota in the PCPA and control groups was performed. The results indicated that 55 bacteria in fecal samples differed between the two groups at all the phyla levels: class, order, family, genera, and species (Figures 2F–I, Supplementary Figures 1–3) at the genus level. The relative abundances of *Klebsiella*, *Escherichia-Shigella*, *Family_XIII_AD3011_group*, *UCG-009*, *Desulfovibrio*, *Enterococcus*, and *[Eubacterium]_coprostanoligenes_group* were significantly elevated in PCPA mice compared with the control group. In contrast, the relative abundances of *Rikenella, Lachnospiraceae_UCG-006, Rikenellaceae_RC9_gut_group, Leucobacter, Paenochrobactrum*, and *Peptococcus* were reduced in PCPA mice than in the control group (Supplementary Figure 2).

Furthermore, the KEGG pathway database was utilized to determine changes in functional composition. At Level 1, we observed that the higher abundance difference in the PCPA group was associated with metabolism, human disease, etc., (Figure 2J). At Level 2, the proportion of sequences related to the circulatory system, signaling molecules and interaction, cellular community eukaryotes, and substance dependence was elevated in the control group (Supplementary Figure 3). At Level 3, four KEGG pathways were significantly enriched in the PCPA group, including phenylpropanoid biosynthesis, tropane, piperidine, pyridine alkaloid biosynthesis, endocytosis, and isoquinoline alkaloid biosynthesis (Figure 2K, Supplementary Figure 4).

### Para-chlorophenylalanine treatment reduced acetic acid in the blood and hippocampus

**FIGURE 3:**
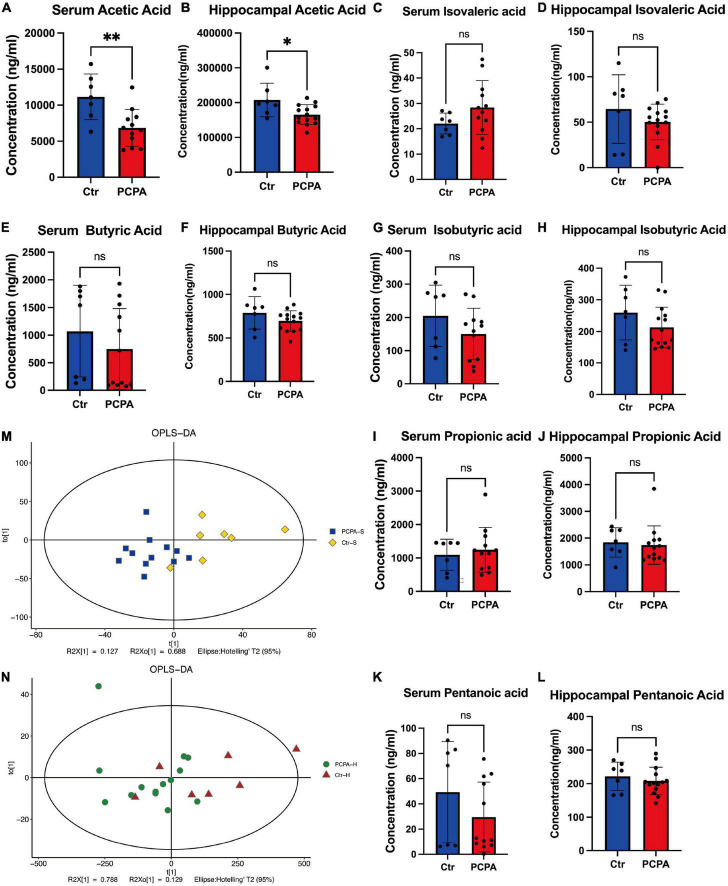
The levels of short-chain fatty acids (SCFAs) in the blood and hippocampus. **(A–L)** Comparison of the SCFA levels in the hippocampus and the blood between two groups. **(M,N)** OPLS-DA plot. Data are presented as the mean ± SEM. *P < 0.05; ^**^P < 0.01; ns, not significant.

Since many researchers have fixated on the relationship between short-chain fatty acids and NCDs (Van De Wouw et al., 2018; Dalile et al., 2019; Kaur et al., 2020), we attempted to open this black box by considering the mediating role of SCFAs. We performed LC-MS analysis to quantify the levels of short-chain fatty acids. The concentration of acetic acid within the blood and hippocampus of the PCPA group was significantly reduced compared with the control group (Figures 3A,B). However, there was no significant difference in the levels of isovaleric acid, butyric acid, isobutyric acid, propionic acid, and pentanoic acid between the serum and hippocampus (Figures 3C–L). In order to exhibit our results clearly, the OPLS-DA analysis was performed (Figures 3M,N).

### Correlation of short-chain fatty acids levels with dysbiosis and neurocognitive disorders

**FIGURE 4:**
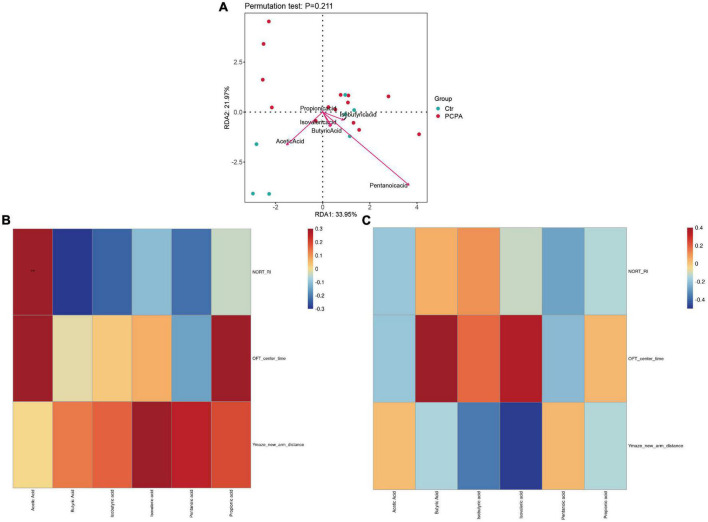
Correlation of short-chain fatty acids (SCFAs) levels with dysbiosis and cognitive impairment. **(A)** Correlations between the SCFAs and gut microbiota. **(B)** Heatmap of corrections between SCFAs and behavioral tests of the para-chlorophenylalanine (PCPA) group. **(C)** Heatmap of corrections between SCFAs and behavioral tests of the Control group.

Furthermore, we performed Spearman’s rank correlation analysis to test correlations between behavioral tests and SCFAs abundance to advance our understanding of the connection between NCDs and the gut microbiota (Figures 4B,C). The result showed that the levels of acetic acid in the hippocampus of the PCPA group were associated with the recognition index of the open field test. In addition, we conducted a redundancy analysis (RDA) of bacterial communities at the genus level colonized by the microbiota. As shown in Figure 4A, RDA revealed that 21.97 and 33.95% of the variance at the first and the second RDA axes were explained, respectively.

## Discussion

Based on the effect of 5-HT depletion, PCPA injection has induced animal models of insomnia in the past decades for a long time (Prospero-García et al., 1993; Shi et al., 2019; Du et al., 2020). However, a decrease in the serotonin level also causes cognitive deterioration. Previous studies indicated that serotonergic neuron has a primary role in cognition (Sun et al., 2021; Shine et al., 2022). The alternation of the 5-HT neuron system significantly affects NCDs. Although the mechanism involved in NCDs is unclear, dysfunctions of multiple 5-HT pathways in the brain have been observed in various diseases (Upton et al., 2008). The administration of PCPA in mice causes serotonin depletion, which is associated with behavioral deficits. Furthermore, recent investigations on the relationship between gut microbiota and cognition, including NCDs related to age, could be reversed by gut flora transplantation. Similarly, such relationship was observed in other diseases (Boehme et al., 2021; Qian et al., 2022; Thu Thuy Nguyen and Endres, 2022).

This article aimed to shed light on the temporal relations between PCPA treatment and NCDs. Therefore, the administration of PCPA significantly depleted serum 5-HT levels in mice. Consequently, the PCPA group exhibited a decreased considerably travel time in the new arm of Y-maze and a reduced recognition index in the NORT, indicating the occurrence of NCDs, consistent with the results of studies of PCPA-induced NCDs among mice (Ngoupaye et al., 2020; Li et al., 2022a). Interestingly, a decrease in time spent in the central area in OFT demonstrated depression-like behaviors in PCPA-treated mice. Simultaneously, the composition of gut flora was different between the PCPA and the control groups, which indicated that dysbiosis was also involved in PCPA-induced NCDs. A significant reduction in the relative abundance of acetate-producing bacteria such as *Lactobacillus* and *Parabacteroides* was observed in the PCPA group.

Furthermore, after the PCPA treatment, it was observed that the level of acetic acid was significantly reduced in the blood and hippocampus than in the control mice. Increasing evidence indicates that acetate depletion played a vital role in various NCDs. For instance, Zheng et al. (2021) observed that acetate, as the primary production of dietary fiber of gut flora metabolite, could protect the NCDs by regulating hippocampal synaptophysin (SYP). Therefore, Spearman’s rank correlation analysis determined the association between the results of behavioral tests and SCFAs. A significant correlation was also observed between the recognition index of NORT and acetic acid in the PCPA group. Depression-like behaviors demonstrated by OFT also correlated with a decreased level of acetate (though non-significantly). Furthermore, according to RDA, levels of acetic acid and pentanoic acid were associated with the gut microbiota. Therefore, we speculate that cognitive dysfunction and depression-like behaviors induced by the PCPA treatment in mice may be related to dysbiosis and SCFAs.

Linear discriminant analysis effect size analysis revealed 19 features that significantly varied between the two groups (PCPA, 11; control, 8). This reasoning leads us to expertise with a positive relationship between PCPA-related NCDs and dysbiosis. Qu et al. (2022) observed that LPS-containing taxa, such as *Proteobacteria*, *Gammaproteobacteria*, *Enterobacteriaceae*, and *Escherichia–Shigella*, were positively associated with NCDs. Lipopolysaccharide (LPS), an endotoxin generated by G-bacteria, has previously been identified its relationship with the production of gut inflammation cytokines (Gao et al., 2022; Nguyen et al., 2022). On the contrary, they also determined that SCFA-producing bacteria, which *Prevotella* represents, decreased significantly in patients with NCDs. Surprisingly, the LPS-containing bacteria mentioned above ranked in the top 3 among the 11 features in the PCPA group, and *Prevotella* was also observed in the control group.

These findings are supported by the reports from Ngoupaye et al. (2020) and Riga et al. (2020) that PCPA-treated mice revealed depression-like behavior. In the above studies, the authors reported that antidepressants could decrease the impact of PCPA on NCDs. However, such a reversible effect is incomplete. Vortioxetine, an antidepressant mentioned above, exerts its influence partially on behavioral deficits as a 5-HT receptor agonist. However, acute administration of vortioxetine (0.1 mg/kg) failed to improve the spontaneous alternations of mice. Although a higher dosage of vortioxetine (3.0 mg/kg) enhances performance, the total number of responses in spontaneous alternations did not improve. Our findings may be interpreted from another perspective. As hypothesized, the results depicted that a conceptual understanding of PCPA-induced depression was also related to the gut microbiota and acetate. Thus, even though we did not find strong support for the relationship, it is too early to conclude that dysbiosis could not play an enormous role in this process.

Besides depression-like behavior, PCPA promotes NCDs, including learning and short-term memory lesions. Hritcu L. et al. (2007) and Teixeira et al. (2018) discovered that PCPA treatment significantly reduces spontaneous alternation percentage in Y-maze, aligning with our findings. Although the effects of the 5-HT receptor agonist on the PCPA-treated mice remain unclear, the reduction in SCFA-producing bacteria and SCFA levels in the brain, in turn, could lead to NCDs (Liu et al., 2022b). Therefore, there is a reason to believe that the microbiome depends on PCPA-evoked NCDs. However, previous studies showed that dysbiosis and SCFA levels also might be related to insomnia induced by PCPA (Wang et al., 2020; Si et al., 2022a). PCPA-treated mice did not have insomnia in our study. In this study, we choose PCPA methyl ester as the 5-HT depletor. Compared with the insolubility of PCPA, PCPA methyl ester is very soluble in both PBS and saline (Murray et al., 2015; Du Jardin et al., 2017), which directly affects the absorption rates of the drug. The PCPA methyl ester solution could be absorbed by rodents much easier than PCPA suspension, which leads to the rapid elimination of PCPA methyl ester. In the previous studies (Murray et al., 2015), the animal model of insomnia was induced by PCPA methyl ester under 800 mg/kg, and there was no evidence showing that PCPA methyl ester could also cause insomnia under 300 mg/kg. Hence, we suppose that insomnia did not appear under a low dose of PCPA methyl ester in mice due to its rapid absorption and elimination. It can be inferred that dysbiosis and SCFA levels might not be related to insomnia induced by PCPA in this study (Supplementary Figure 5).

Our study has strengths, including the unique perspective on performance alternations in PCPA-treated mice. It is the first to demonstrate the effects of the SCFAs and main acetate on such disorders. However, our study also has certain limitations that can be improved. First, additional research is required to investigate whether applying direct acetate to PCPA-treated mice may ameliorate NCDs induced using PCPA. Second, the alternation of acetate levels in the hippocampus is related to intestinal permeability or only to the increased intestinal barrier. Therefore, a future empirical study is required.

## Conclusion

In this article, multiple studies were conducted to identify the potential mechanisms of PCPA-induced NCDs associated with dysbiosis in mice. Our findings suggest that the promotional effects of PCPA treatment on dysbiosis could lead to a lower concentration of acetate in the hippocampus, associated with NCDs. Although PCPA has not been applied in clinical practice due to its adverse effects, the findings of this study provided a new perspective on cognitive impairments due to PCPA and PCPA-based animal models.

## Data availability statement

The datasets presented in this study can be found in online repositories. The names of the repository/repositories and accession number(s) can be found below: https://www.ncbi.nlm.nih.gov/, SUB11975467.

## Ethics statement

The animal study was reviewed and approved by Animal Care and Use Committee of Tongji Hospital, Tongji Medical College (No. TJ0803).

## Author contributions

All authors listed have made a substantial, direct, and intellectual contribution to the work, and approved it for publication.
