# Supplementary material for: Gut microbiota-generated short-chain fatty acids are involved in para-chlorophenylalanine-induced cognitive disorders
Source: Front Microbiol. 2022 Nov 7;13:1028913. doi: 10.3389/fmicb.2022.1028913 (PMC9676499; doi:10.3389/fmicb.2022.1028913)
Supplement: Supplementary file 1 [file Data_Sheet_1.ZIP › PCPA NCDs.Supplementary_Material 2.docx]

Supplementary Material

#
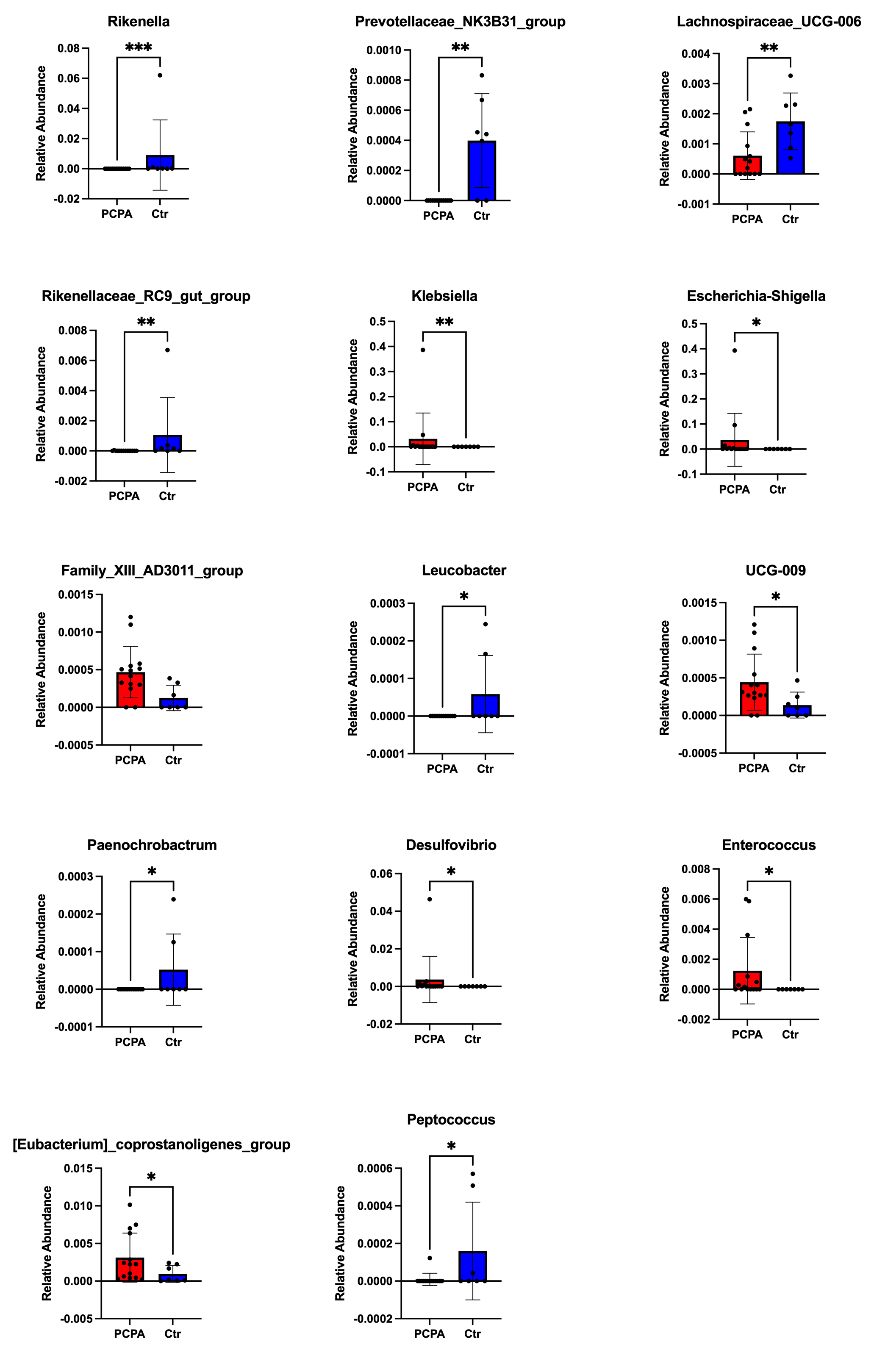
Supplementary Figures

**Supplementary Figure 1.** The relative Abundance of gut microbiota at the genus level.

**Supplementary Figure 2.** The relative Abundance of gut microbiota at the species level.


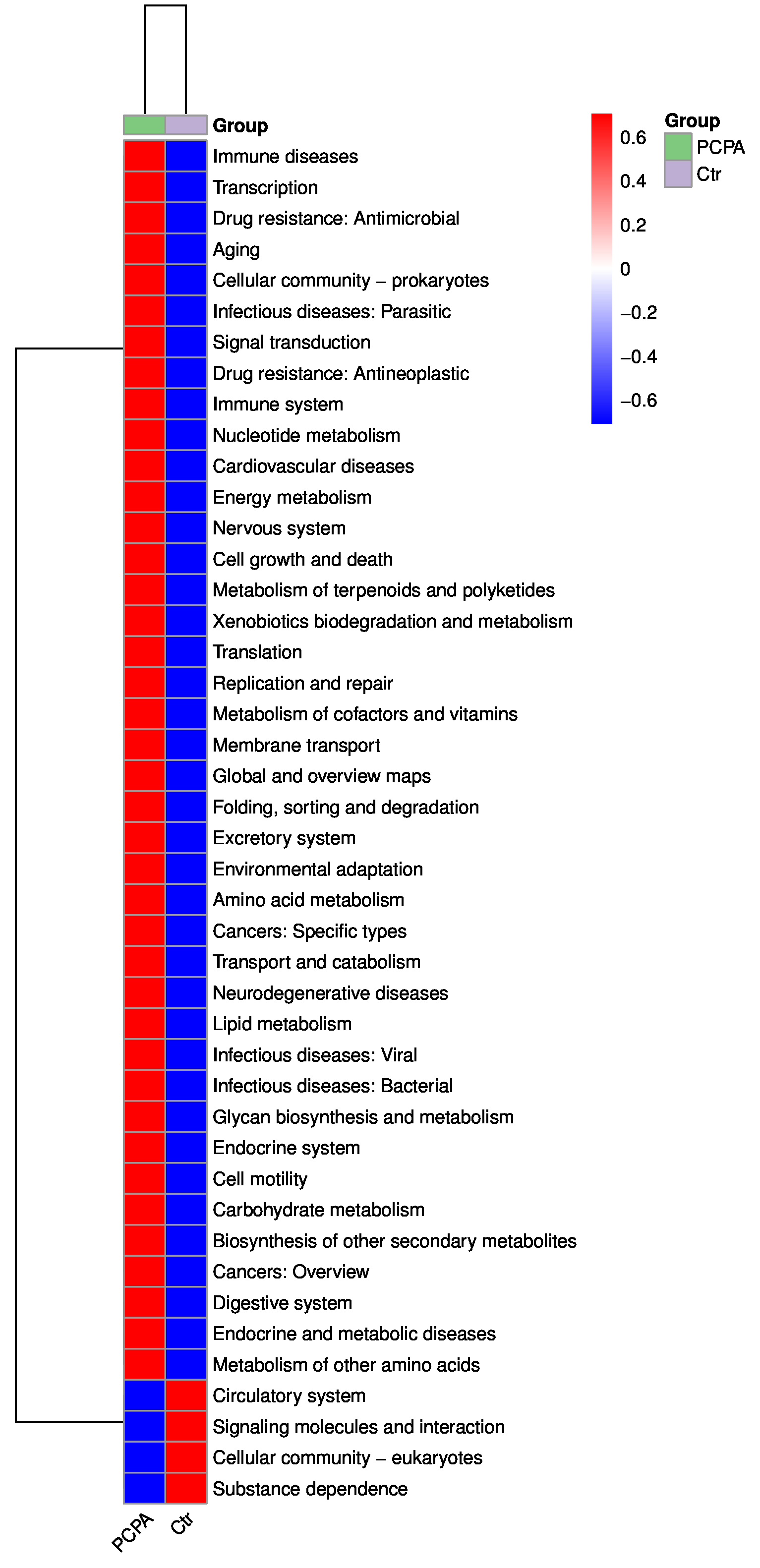


**Supplementary Figure 3.** Heatmap of KEGG at level 2.

**Supplementary Figure 4.** Heatmap of KEGG at level 3.


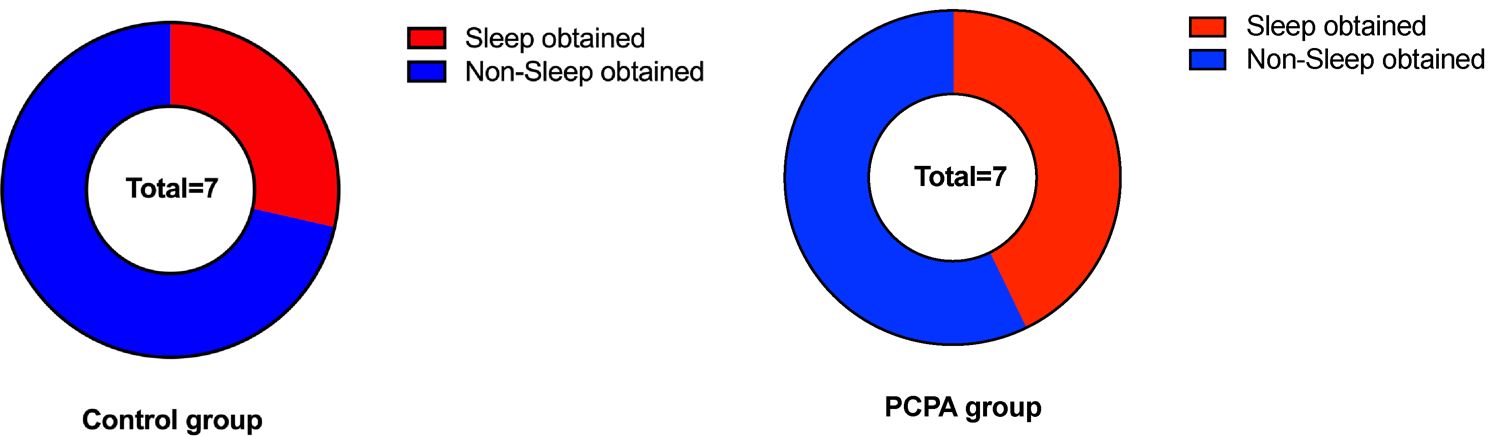


**Supplementary Figure 5.** Effect of PCPA methyl ester on the insomnia of mice.

.
